# Late results of a randomized trial on the role of mild hypofractionated radiotherapy for the treatment of localized prostate cancer

**DOI:** 10.7150/jca.37825

**Published:** 2020-01-01

**Authors:** Petros Alexidis, Sotirios Karatzoglou, Dimitris Dragoumis, Konstantinos Drevelegas, Ioannis Tzitzikas, Konstantinos Hatzimouratidis, Ioannis Chrisogonidis, Aris Ioannidis, Iason Nikolaos Katsios, Paul Zarogoulidis, Konstantinos Sapalidis, Charilaos Koulouris, Nikolaos Michalopoulos, Dimitrios Giannakidis, Zoi Aidoni, Varbara Fyntanidou, Aikaterini Amaniti, Konstantina Boniou, Isaak Kesisoglou, Anastasios Vagionas, Konstantinos Romanidis, Panagoula Oikonomou, Alexandru Marian Goganau, Savas Petanidis, Elena Maragouli, Christoforos Kosmidis

**Affiliations:** 1 Department of Radiation Oncology, “ Interbalkan European Medical Center” Thessaloniki, Greece.; 2 Neurosurgical Department, “G. Papanikolaou” General Hospital, Thessaloniki, Greece.; 3 Radiology Department, Everlight Radiology, U.K.; 4 Department of Radiation Oncology, AHEPA University Hospital of Thessaloniki, Faculty of Medicine, School of Health Sciences, Aristoteleion University of Thessaloniki, Greece.; 5 Department of Urology, Papageorgiou hospital of Thessaloniki, Faculty of Medicine, School of Health Sciences, Aristoteleion University of Thessaloniki, Greece.; 6 Department of Radiology, AHEPA University Hospital of Thessaloniki, Faculty of Medicine, School of Health Sciences, Aristoteleion University of Thessaloniki, Greece.; 7 3rd Department of Surgery, “AHEPA” University Hospital, Aristotle University of Thessaloniki, Medical School, Thessaloniki, Greece.; 8 Ιntensive Care Unit (ICU), “AHEPA” University Hospital of Thessaloniki, Faculty of Medicine, School of Health Sciences, Aristoteleion University of Thessaloniki, Greece.; 9 Anesthesiology Department, “AHEPA” University Hospital, Aristotle University of Thessaloniki, Medical School, Thessaloniki, Greece.; 10 Radiotherapy Department, “Theageneio” Anti-Cancer Hospital, Thessaloniki, Greece.; 11 Oncology Department, General Hospital of Kavala, Kavala, Greece.; 12 Second Department of Surgery, University Hospital of Alexandroupolis, Medical School, Democritus University of Thrace, 68100 Alexandroupolis, Greece.; 13 General Surgery Clinic 1, University of Medicine and Pharmacy of Craiova, Craiova County Emergency Hospital, Craiova, Romania; 14 Department of Pulmonology, I.M. Sechenov First Moscow State Medical University; Moscow, 119992, Russian Federation.; 15 Oncology Department, University of Thessali, Larissa, Greece.

**Keywords:** hypofractionation, early, late toxicity, prostate cancer.

## Abstract

**Background:** Prostate cancer is considered to be highly sensitive to changes in radiation therapy dose per fraction, specifically to hypofractionation. An increase in the fractionation dose could cause a higher increase to the prostate than to the normal tissues leading to better disease control with less toxicity. Here we present the results of a randomized trial comparing mild hypofractionation to conventional fractionation after a median of 3,6 years follow up. **Patients and Methods:** 139 patients were randomized to receive either hypofractionated radiotherapy with 2,25 Gy/fr to a total of 72 Gy (arm 1) or conventionally fractionated treatment with 2Gy/fr to a total of 74 Gy (arm 2). 72 patients were assigned to arm 1 and 67 to arm 2. **Results:** After a median follow up of 3,6 years, 23 patients (31,9%) from arm 1 developed grade≥ 2 acute genitourinary toxicity and 21 (31,3%) from arm 2 (p=0,79). The corresponding values from gastrointestinal were 15 (20,8%) and 12 (17,9%) (p=0,6). For late toxicity from GU, 8 patients (11,1%) developed grade≥ 2 symptoms in arm 1 and 7 (10,4%) in arm 2 (p=0,92). late GI toxicity grade≥ 2 was observed in 8 (11,1%) patients in arm 1 and 8 (11,9%) in arm 2 (p=0,88). In multivariate analysis, hormone therapy was significantly associated with late GI events, while acute toxicity from both GU and GI was a prognostic factor of late adverse reaction. **Conclusion:** No difference in the toxicity profile could be identified between hypofractionation and conventional fractionation. Our schedule of 2,25Gy/fr seems safe and tolerable by the patients with acceptable rates of acute and late toxicity.

## Introduction

Radiation therapy is one of the most important and commonly used treatments for prostate cancer. Every year there are 164000 new prostate cancer patients in the US and many of those will require definitive treatment with radiation. Delivering an effective dose to the disease site is of great importance since dose escalation has been shown to offer a benefit to disease control.^1^-^6^ Conventionally, 1,8-2 Gy/fr are being used to a total dose of 75,6 - 81 Gy 7-9, which requires up to 45 fractions to be delivered. This is inconvenient for the patients and leads to excessive usage of medical services. Being able to deliver sufficient dose to the prostate by decreasing the total number of fractions is an important goal which could be achieved with hypofractionation. The distinct biology of prostate cancer makes this disease quite sensitive to altered fractionation, meaning that a rise in the dose per fraction would achieve a higher dose to the prostate cancer cells compared to the dose delivered to the normal tissue. The hypothesis is that with hypofractionation we can deliver a higher biologically equivalent dose (BED) to the prostate compared to normal tissue, thus leading to shorter treatment duration without an increase in the toxicity.

We previously published preliminary results of our study 10, comparing conventional fractionation to hypofractionation with 2,25 Gy/fr and no difference in the toxicity profile was observed between groups. Here we present the updated results for late toxicity 10.

## Patients and methods

### Patients

The study was approved by the investigational review board (IRB) of “G. Papageorgiou” University Hospital. In this randomized trial we included patients with localized prostate cancer (cT1c-cT3bN0M0), that were treated with either conventional fractionation of 2 Gy/fr in 37 fractions or 2,25 Gy/fr in 32 fractions to the prostate only with or without the seminal vesicles with intensity modulated radiation therapy (IMRT). Patients participating in the trial should be between 40 and 85 years old, with a biopsy proven prostate cancer, performance status 0-2 and PSA level no more than 40 ng/ml. Patients with a history of prostatectomy (suprapubic or transurethral), bladder cancer and/or transurethral resection of bladder tumor (TURBT), inflammatory bowel disease, hip replacement, previous irradiation of the pelvis and patients with a pathological uroflowmetry were excluded from the study. Additionally, we did not include patients with a calculated risk of lymph node involvement ≥5% 11, those with T3 disease and GS ≥8, T3 disease and PSA>10 ng/ml, GS 8-9 and stage T3 or T4 or PSA >10 ng/ml. All patients were staged with digital examination, prostate biopsy, PSA evaluation, and CT of the pelvis and abdomen. Pelvic MRI and bone scan were prescribed for patients with T3-T4 stage, PSA >20 ng/ml or GS 8-9 or for those with symptoms. This trial was approved by the medical ethics committee of Aristotle University of Thessaloniki. All patients provided written informed consent.

### Procedures

An LHRH analogue combined with initial anti androgen to reduce testosterone flair was given as androgen deprivation therapy (ADT) 2 months before initiation of radiotherapy. ADT duration was 6 months for patients with intermediate risk disease and 2-3 years for patients with high risk disease according to the physician's discretion. CT simulation was performed by acquiring a 3 mm slice CT of the pelvis from L4 vertebra up to the ischial tuberosities. The patient was instructed to use an enema the previous day and drink 500 ml of water 45 minutes before CT scan. The prostate with or without the seminal vesicles (SV) (based on risk of SV involvement) 12 were delineated and patients were divided into three groups. Patients with risk of SV involvement of less than 10% were included in group 1 and only the prostate was treated to the maximum dose, while the second group included patients with risk of 10-25% (SV group 2) and the prostate together with the proximal 1 cm of the SV was treated to the maximum dose while the rest of the SV was treated with a lower dose of 56 Gy. The third group (SV group 3) included patients with a risk of >25%. In this group the prostate and the proximal 2 cm of the SV were treated to the maximum dose unless the SV were involved where in that case the maximum dose was delivered to the whole SV. Organs at risk included the femoral heads, penile bulb, bladder, bowel bag and rectum and mandatory dose constraints for these organs were defined. A planning target volume (PTV) of 1 cm to all directions and 5 mm posteriorly was used. The patients' position was evaluated and corrected by everyday KV imaging and cone beam CT once weekly. Patients in the first arm received 72 Gy with 2,25 Gy/fr, 5 days per week (Monday to Friday) and patients in the second arm received 74 Gy with 2 Gy/fr 5 days per week. Biologically equivalent doses were calculated assuming a/b ratio of 1,5 (BED=180 Gy in arm 1 and 172,7 Gy in arm 2). All patients received treatment with VMAT technique. Randomization was performed by a random number generator using a web-based application 12, 13.

### Outcomes

Acute toxicity was defined as an event that manifested during RT or within the first 3 months after the end of treatment and late toxicity as any event developing after the first 3 months. Both toxicities were evaluated according to the RTOG scoring system (physician completed forms)^12^, ^14^, every week during RT and on weeks 11, 15 and 19 from start of RT for the acute phase. Late toxicity was evaluated every 6 months for the first year after the end of RT and then annually. Quality of life was evaluated by patient reported questionnaires.^15^ Before start of RT, baseline scores were collected for each patient and a mean baseline value was calculated for the whole cohort. For the acute phase, patients were evaluated again on weeks 4, 11 and 19 from start of RT and on months 6, 12 and 24 after the end of RT for the late phase. The change in quality of life for each patient was determined by subtracting the new score from the baseline value.

### Statistical analysis

The GU and GI toxicity events (RTOG scale) were grouped into two categories, grade <2 and grade ≥2 and we used the Kaplan Meier method to analyze them. The curves were compared with Log Rank test and Cox regression analysis was used to identify any possible correlation between baseline patient and treatment factors and toxicity. The variables included in the model were age, seminal vesicles invasion group, T stage, Gleason score, PSA, percentage of positive biopsy cores, hormone therapy, risk group, acute GU and GI toxicity. The change in mean values from self-assessment questionnaires for each group were compared by T test or non-parametric Man Whitney U test. Statistical significance for all tests was set at 0,05. Descriptive statistics were used to analyze patient and treatment characteristics and SPSS version 25 for the statistical analysis.

## Results

**Figure 1:**
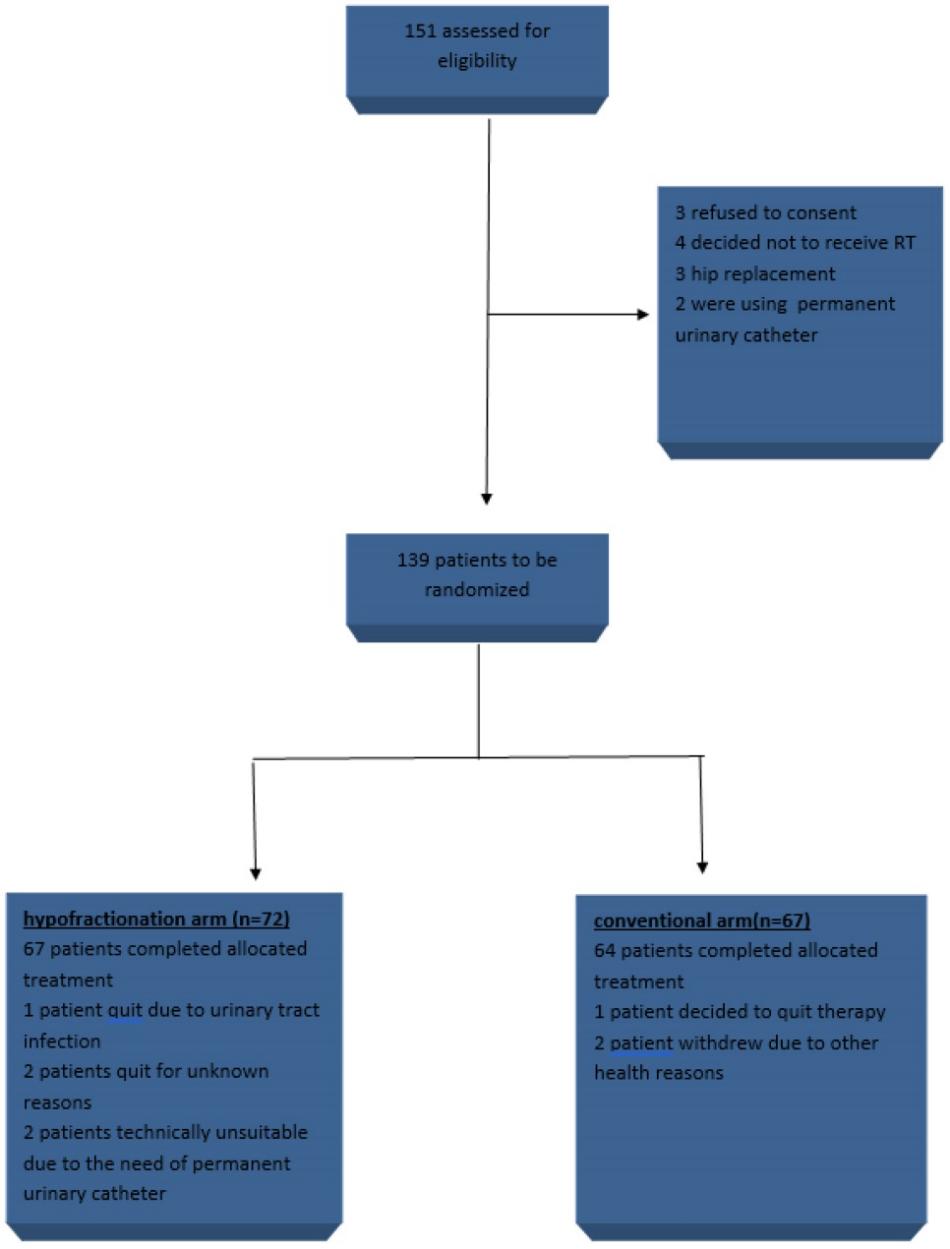
trial profile.

From 2015 to 2016 139 patients were included in the study, 72 in arm 1 (HRT) and 67 in arm 2 (CRT) (figure 1). Baseline patients and treatment characteristics were equally distributed between arms and are summarized in table 1. Mean age was 70,3 years, 38 (27,3%) patients belonged to the low risk group, 52 (37,4%) to intermediate and 49 (35,3%) to high. 96 (69,1%) patients received ADT, from which 10 belonged to the low risk group, 43 to intermediate and 43 to high. When grouping patients according to risk of seminal vesicles involvement, 86 (61,9%) had a risk of <10% for disease in the SV, 44 (31,7%) had 10-25% and 9 (6,5%) had >25% chances of SV involvement. In CRT arm 64 completed allocated treatment, 1 patient decided to quit treatment and 2 withdrew due to other health reasons. In the HRT, 67 patients completed treatment, 1 quit due to urinary tract infection, 2 quit for unknown reasons and 2 were unsuitable to continue in the study due to the need of permanent urinary catheter.

**Figure 2:**
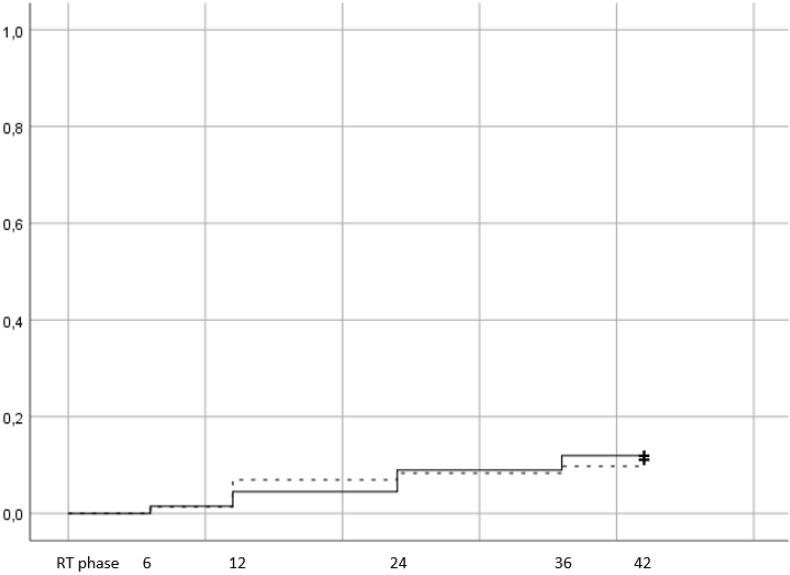
Cumulative incidence of late grade >=2 GI toxicity (Log rank p value= 0,88). Time in months from the end of RT. RT phase represents the treatment period, plus 3 months after the end of RT and corresponds to the acute toxicity evaluation.

There were 16 patients in total that developed late GI ≥2 toxicity, 8 in arm 1 (11,1%) and 8 in arm 2 (11,9%). The results are presented with Kaplan Meier curves in figure 2.

Log rank test did not reveal a statistically significant difference between arms (p=0,88). Of the 16 patients that developed late GI toxicity grade ≥2, 8 had developed acute GI as well (4 in the HRT and 4 in the CRT). Men that were treated with ADT were more likely to suffer from late GI toxicity (14 out of 16 patients in total), while the distribution of toxicity was uniform between sv groups (11% in sv group 1, 15% in group 2 and 11,1% in group 3). After testing for potential factors that could affect toxicity, only acute GI grade ≥2 was significantly correlated with late GI grade ≥2 toxicity (p=0,009) on univariate analysis (table 2).

**Figure 3:**
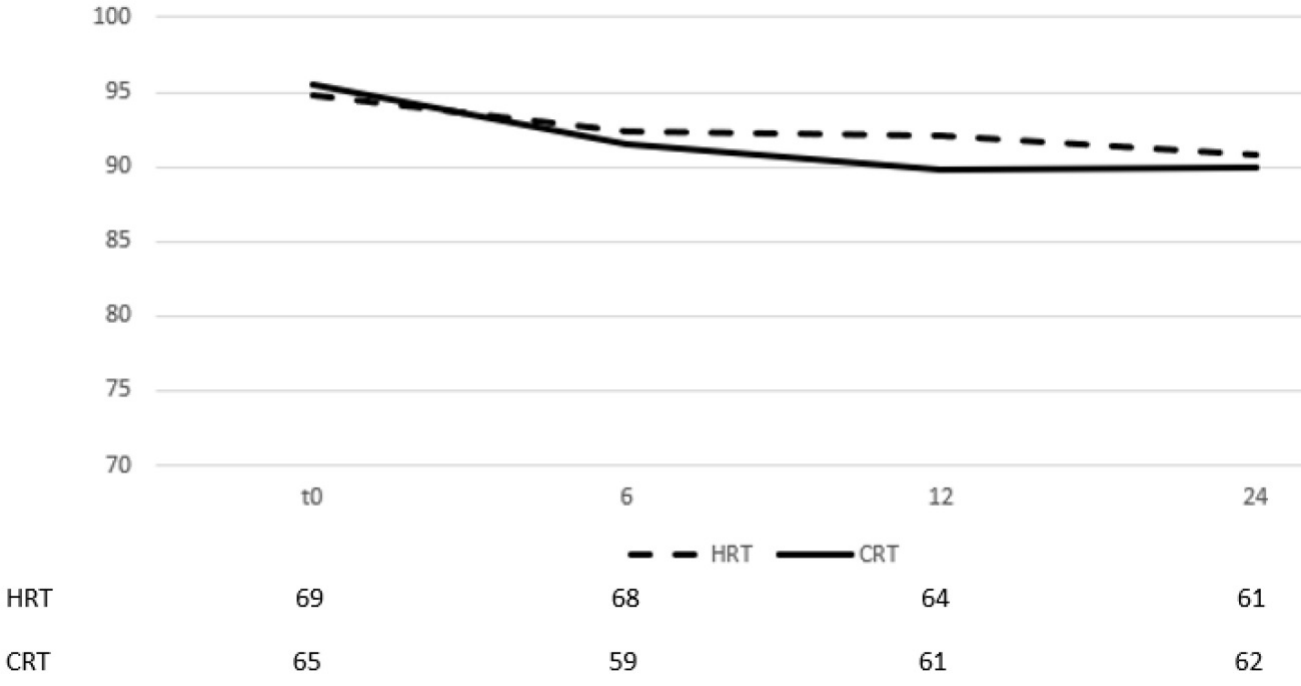
Change of means in relation to time for GI quality of life assessment. Time in months after the end of RT, t0 represents group baseline values before start of treatment

On multivariate analysis, acute GI toxicity (p=0,001) and hormone therapy (p=0,001) were prognostic factors of late GI toxicity. The self-assessment questionnaires evaluating quality of life from GI showed no difference between groups in all three time points (table 3) (there was only a marginal significance on month 24). By month 6 after the end of RT, most patients had recovered from their symptoms and the mean scores for each group had returned to slightly lower values than those documented before start of RT (figure 3).

The scores remained relatively stable between months, 6, 12 and 24 with very low volatility, documenting a stable quality of life status.

**Figure 4:**
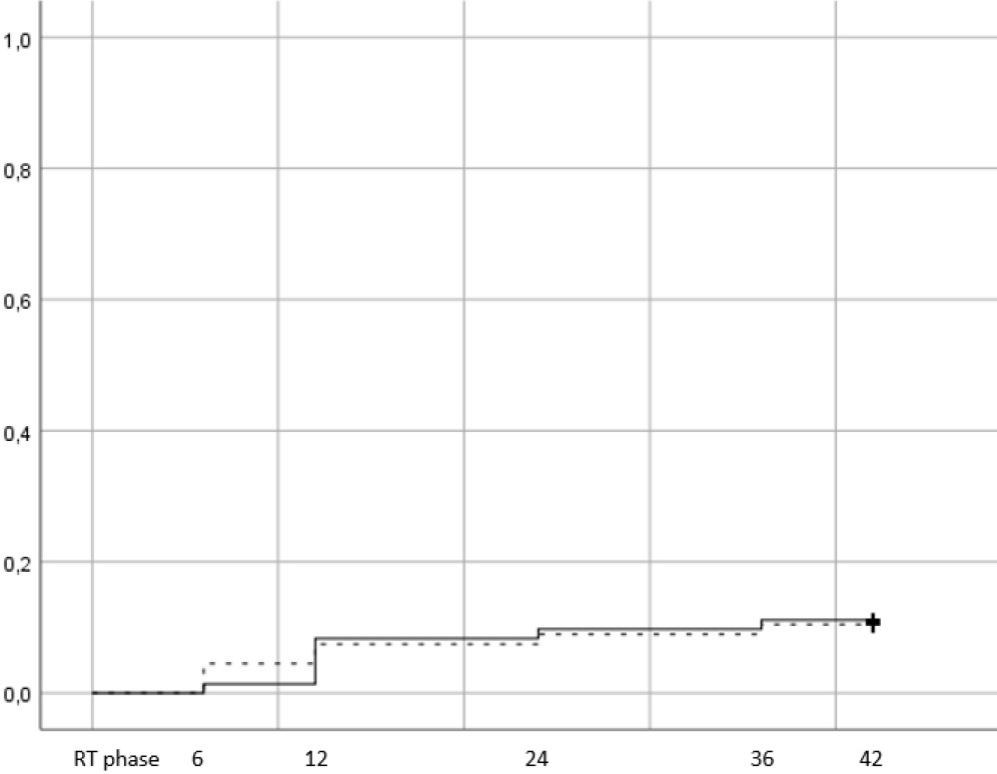
Cumulative incidence of late grade >=2 GU toxicity (Log rank p value= 0,79). Time in months from the end of RT. RT phase represents the treatment period, plus 3 months after the end of RT and corresponds to the acute toxicity evaluation.

There were 15 events of late GU grade ≥2 toxicity, 8 in the hypofractionation arm (11,1%) vs 7 (10,4%) in the conventional (figure 4, p=0,79). 12,7% of patients with age <70 years developed late GU toxicity vs 10,5% for patients ≥70 years.

**Figure 5:**
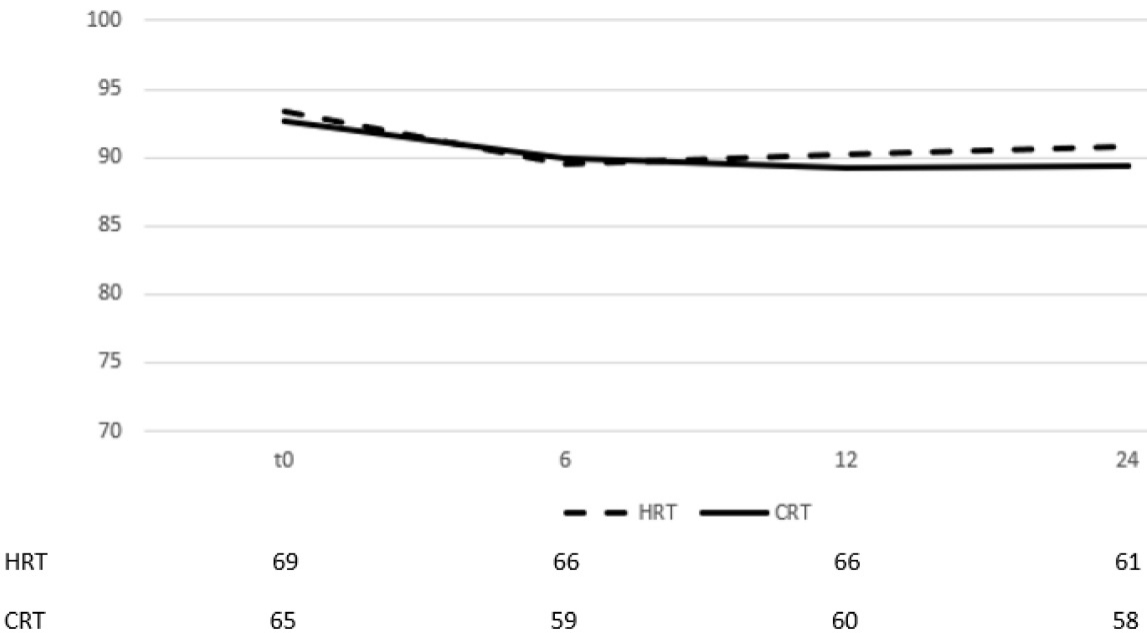
Change of means in relation to time for GU quality of life assessment. Time in months after the end of RT, t0 represents group baseline values before start of treatment.

When men were stratified according to hormone therapy treatment, 12% of those that did not receive ADT presented with late GU vs 12,1% of those that received. Of the 15 patients, 9 had developed acute GU grade ≥2 (5 in the HRT arm and 4 in CRT). Cox regression univariate and multivariate analysis showed that the only significant prognostic factor of late GU was acute GU grade ≥2 (table 4 p=0,045). Quality of life assessment from GU showed an improvement during the late phase evaluation (figure 5), with no statistically significant difference between time points and stable score values across timepoints (table 3).

## Discussion

This is a randomized trial comparing conventional fractionation to mild hypofractionation. Our schedule of 2,25 Gy/fr to a total of 72 Gy is safe and easily tolerable by the patients. We found no statistically significant differences between arms in the toxicity profile of GU and GI, or quality of life. Late GU and late GI were significantly associated with acute toxicity, while late GI toxicity was also correlated with hormone use on multivariate analysis. In our previous publication of acute toxicity evaluation, we observed 23 (31,9%) events of acute GU grade ≥2 toxicity in arm 1 and 21 (31,3%) in arm 2 with no difference between HRT and CRT (p=0,79). Toxicity developed in 25 of patients receiving ADT vs 17 not receiving and older patients (>70 years) were more likely to develop such an event (19 patients in <70 years old group vs 25 in ≥ 70 years). On cox regression analysis none of the aforementioned factors was correlated with acute grade ≥2 toxicity from GU. Univariate and multivariate analysis did not show a significant association with any of the other baseline factors as well. Acute GI toxicity grade ≥2 was observed in 15 (20,8%) patients in arm 1 and 12 (17,9%) patients in arm 2. 16 (19,5%) patients in SV group 1 developed grade ≥2 toxicity vs 10 (25%) and 1 (11,1%) for groups 2 and 3 respectively. On univariate cox regression analysis, no factors were associated with toxicity and the difference between arms was not significant (p=0,6). Adverse reactions in the hypofractionated arm peaked on week 5 and on week 7 for conventional fractionation. These weeks represent the competition of the treatment for each arm and by then, the full dose has been delivered to the prostate leading to higher toxicity rates. At the peak there was no statistical difference between arms (p=0,91) for GU (p=0,91) and GI (p=0,64). By week 19, patients had recovered almost completely, with percentages returning to the values observed during the first week of treatment. The mean scores from the self-assessment questionnaires for acute GU and GI toxicity, showed a drop during the first month from start of RT for both arms and an increase was seen on months 3 and 5 with the values remaining relatively stable. After comparing the difference from the baseline score no significant differences were observed between groups for all the three time points.

A correlation between age and late GU toxicity has been previously described.^16^, ^17^ Pollack et al found that late GU toxicity was significantly higher for patients above 67 years old in the hypofractionated arm, while in HYPRO trial age >70 was associated with side effects for both GU and GI irrespective of treatment group. Just like acute side effects mentioned earlier, no such correlation was identified for late events either. 7 (12,3%) and 5 (8,8%) of patients in the <70 years old group developed grade ≥2 late toxicity from GU and GI compared to 8 (9,8%) and 11 (13,4%) for patients ≥70 years suggesting that our schedule is safe across all ages.

Some studies have found an association between the length of seminal vesicles being treated and GI toxicity.^16^, ^18^-^20^ The rationale is that when a bigger portion of the seminal vesicles is irradiated, this increases the percentage of the rectum volume that is exposed to the high dose. This was further supported by a meta-nalysis^19^ of 9 phase 3 trials comparing HRT to CRT, which showed that patients group with <76% full seminal vesicles treated to the high dose vs ≥76% significantly influenced the incidence of GI toxicity with HRT. We found no correlation between sv group and GI toxicity but this result should be interpreted with caution. The size of our cohort is relatively small and there were few patients in the SV group 3. Had the distribution between SV groups been more uniform, a possible difference in the toxicity profile might have been observed.

The toxicity documented in our study is comparable, and in some cases lower, to that observed in other randomized studies. Aluwini et al 16 recruited 820 men and randomized them to either HRT of 64,6 Gy (3,4 Gy/fr) or conventional treatment of 78 Gy (2 Gy/fr). The late toxicity grade ≥2 observed was 17,7% and 39% from GI and GU respectively in the conventional treatment group vs 21,9% and 41,3% in the hypofractionated. Pollack et al 17 observed GU and GI toxicity rates of 37,9% vs 39,1% and 22,5% vs 18,1% for CRT and HRT respectively. Two other randomized studies found higher toxicity 21, 22 as well but it should be underlined that 3DCRT was also permitted apart from IMRT. A better toxicity profile for both hypofractionated and conventional treatment was observed in CHHIP trial.^23^, ^24^ After enrolling 3216 patients and randomizing them to either 74 Gy of CRT or two schedules of hypofractionation (60 Gy in 20 fractions or 56 in 19 fractions), the late GI toxicity rates grade ≥2 observed were 13,7% (74Gy), 11,9% (60Gy) and 11,3% (57Gy). For late GU the events percentages were 9,1% (74 Gy), 11,7% (60 Gy) and 6,6% (57 Gy). Arcangeli et al 25 also found limited side-effects, with late GU and GI adverse events of 16% and 17% for hypofractionation vs 11% and 17% for conventional RT. The higher toxicity rates observed in some studies could probably be due to the higher BED used. The last years there has been a trend towards increasing the dose which was triggered by the benefit in disease control^1-6^ observed in previous studies and was further facilitated by the broad use of IMRT which led to a significant decrease in the observed toxicity.^7^, ^26^-^29^ Despite the use of newer technologies, higher toxicity was still observed in some cases.^2^, ^30^-^32^ Our schedule of 72 Gy in 32 fractions was well tolerated by the patients and is equivalent of 81 Gy in 1,8 Gy/fr. Currently 75,6 - 81 Gy is considered the standard of care for conventional fractionation, so our HRT plan delivers an efficient dose to the prostate with acceptable toxicity and a shorter total duration. This is particularly important since 81 Gy with standard fractionation of 1,8 Gy/fr would require 45 fractions to be completed while ours requires 13 fractions less.

The limitations of our study are its small size and the relatively small follow up period. We published our late toxicity results at a median follow up of 3,6 years, because there are data in the literature suggesting that most toxicity events occurs the first 3 years after the end of RT.^16^, ^32^ Nevertheless, longer follow up would further support our findings. Another issue which must be addressed is that the patients included in this study had a very good performance status with little comorbidity. It has been reported in previous studies that patients with compromised urinary function, voiding symptoms, or high rates of baseline toxicity grade ≥2 before treatment could develop worse toxicity. 17, 33, 34 Our data support the safety of hypofractionation with 2,25 Gy/fr but the conclusions should not be extrapolated to patients with important comorbidities.

In conclusion, intensity modulated radiotherapy with a hypofractionated regimen of 2,25 Gy/fr can be delivered safely with low rates of acute and late events and a uniform toxicity profile between younger and older patients.

## Figures and Tables

**Table 1:** Patient and treatment factors at baseline.

characteristics	Total n=139 (%)	Conventional fractionation n=67 (%)	Hypofractionation n=72 (%)
Age (mean)	70,3	70,9	69,8
Age categorical			
<70 years	57(41)	27(40,3)	30(41,7)
>=70 years	82(59)	40(59,7)	42(58,3)
Clinical stage			
T1	60(43,2)	28(41,8)	32(44,4)
T2	70(50,4)	36(53,7)	34(47,2)
T3	9(6,5)	3(4,5)	6(8,3)
Gleason Score			
<6	60(43,2)	29(43,3)	31(43,1)
7	61(43,9)	31(46,3)	30(41,7)
8-9	18(12,9)	7(10,4)	11(15,3)
PSA			
<10 ng/ml	84(60,4)	39(58,2)	45(62,5)
10-20 ng/ml	32(23)	17(25,4)	15(20,8)
>20 ng/ml	23(16,5)	11(16,4)	12(16,7)
>50% cores positive			
Yes	55(39,6)	31(53,4)	24(34,8)
No	72(51,8)	27(46,6)	45(65,2)
Hormone therapy			
Yes	96(69,1)	47(73,4)	49(72,1)
No	36(25,9)	17(26,6)	19(27,9)
Risk group			
Low	38(27,3)	18(26,9)	20(27,8)
Intermediate	52(37,4)	28(41,8)	24(33,3)
High	49(35,3)	21(31,3)	28(38,9)
SV involvement probability			
<10%	86(61,9)	44(65,7)	42(58,3)
10-25%	44(31,7)	20(29,9)	24(33,3)
>25%	9(6,5)	3(4,5)	6(8,3)
Type of treatment			
Conventional	67(48,2)	.	.
hypofractionation	72(51,8)	.	.

PSA: prostate specific antigen

**Table 2:** Univariate and multivariate Cox regression analysis for the association of patient and treatment factors with grade >=2 late GI toxicity

	Univariate		Multivariate	
variables	p	OR(CI)	p	OR(CI)
SV invasion risk group				
1 vs 2	0,94	0,92(0,12-7,24)	.	.
1 vs 3	0,85	1,23(0,15-10,3)	.	.
Age (<70 vs >=70)	0,39	1,58(0,55-4,55)	.	.
ADT (yes vs no)	0,18	0,36(0,08-1,6)	0,001	0,064(0,01-0,32)
T stage				
T1 vs T2	0,98	1,03(0,13-8,34)	.	.
T1 vs T3	0,99	1,02(0,13-8,2)	.	.
Risk group				
Low vs intermediate	0,37	0,54(0,14-2,1)	.	.
Low vs high	0,71	0,81(0,27-2,41)	.	.
PSA				
<10 vs 10-20	0,92	0,94(0,26-3,4)	.	.
<10 vs >20	0,7	0,73(0,15-3,61)	.	.
Gleason score				
6 vs 7	0,6	0,7(0,18-2,7)	.	.
6 vs 8-9	0,45	0,59(0,15-2,4)	.	.
Positive biopsy cores (<50% vs >=50%	0,61	0,76(0,27-2,2)	.	.
Acute GI grade ≥ 2 (yes vs no)	0,009	3,66(1,37-9,77)	0,001	18(6,2-55,8)

SV: seminal vesicles, ADT: androgen deprivation therapy, PSA: prostate specific antigen.

**Table 3:** Mean values of quality of life assessment scores and comparison between treatment groups.

	6^th^ month			12^th^ month			24^th^ month		
	HRT	CRT	p	HRT	CRT	p	HRT	CRT	p
Genitourinary	89,5	90	0,14	90,2	89,3	0,52	90,8	89,4	0,33
Gastrointestinal	92,4	91,5	0,71	92,1	89,8	0,7	90,8	89,9	0,051

**Table 4:** Univariate and multivariate Cox regression analysis for the association of patient and treatment factors with grade >=2 late GU toxicity.

	Univariate		Multivariate	
variables	p	OR(CI)	p	OR(CI)
SV invasion risk group				
1 vs 2	0,89	0,86(0,1-6,9)	.	.
1 vs 3	0,8	1,3(0,16-10,84)	.	.
Age (<70 vs >=70)	0,64	0,78(0,28-2,15)	.	.
ADT (yes vs no)	0,98	0,99(0,23-3,1)	.	.
T stage				
T1 vs T2	0,96	1,05(0,13-8,53)	.	.
T1 vs T3	0,93	0,91(0,11-7,41)	.	.
Risk group				
Low vs intermediate	0,88	1,09(0,33-3,6)	.	.
Low vs high	0,49	0,64(0,18-2,28)	.	.
PSA				
<10 vs 10-20	0,6	1,5(0,33-6,74)	.	.
<10 vs >20	0,73	0,71(0,1-5,03)	.	.
Gleason score				
6 vs 7	0,56	0,67(0,17-2,59)	.	.
6 vs 8-9	0,31	0,47(0,11-1,98)	.	.
Positive biopsy cores (<50% vs >=50%	0,4	0,65(0,24-1,78)	.	.
Acute GU grade ≥2 (yes vs no)	0,045	2,9(1,02-8,08)	.	.

SV: seminal vesicles, ADT: androgen deprivation therapy, PSA: prostate specific antigen.
